# The Effect of Stress Management on Occupational Stress and Satisfaction among Midwives in Obstetrics and Gynecology Hospital Wards in Iran

**DOI:** 10.5539/gjhs.v8n9p91

**Published:** 2015-10-29

**Authors:** Mahdi Karimyar Jahromi, Shahnaz Minaei, Sareh Abdollahifard, Majid Maddahfar

**Affiliations:** 1 Research Center for Social Determinants of Health, Jahrom University of Medical Sciences, Jahrom, Iran; 2 Jahrom University of Medical Sciences, Jahrom, Iran; 3 BHOWCO Trading GmbH, Frankfurt am Main, Germany

**Keywords:** stress management, occupational stress, occupational satisfaction

## Abstract

**Introduction:**

Occupational stress is one of the major problems of health care staff, substantially affecting their professional and personal performance. This research has been conducted with the aim of determining the effect of stress management on occupational stress and satisfaction among the Midwives in Obstetrics and Gynecology Hospital wards at Motahari Hospital in Jahrom, Iran 2013-2014.

**Methods:**

This is a Quasi-experimental study of the pre- and post-clinical trials type. The study population included midwives employed in the Obstetrics and Gynecology Hospital wards selected trough census. The samples were categorized into two groups randomly. The intervention group participated in the short-term training workshop of stress management. The studied samples were measured in terms of occupational stress and satisfaction before, right after, and one month after the workshop. Occupational stress measurement was measured by Toft-Anderson occupational stress questionnaire (1981). Similarly, the occupational satisfaction was measured by JDI checklist developed by Stephen Robins (1994). In order to analyze the information, SPSS 16 together with descriptive statistics tests (frequency, percentile, mean, and standard deviation), independent sample t-tests, iterative measurement and Spearman correlation coefficient were used.

**Results:**

A total of 70 people (two 35-person groups) of midwives participated in this study. The findings revealed that there was a significant difference between the mean of scores of occupational stress between the two groups before and after the workshop (p=0.001). There was, however, no significant difference between the scores of satisfactions across the two groups.

**Discussion:**

Training of skills of coping with stress including stress management can be effective in diminishing level of occupational stress. Mitigation of stress without catering for professional, occupational, organizational, and environmental factors would not lead to development of job satisfaction.

## 1. Introduction

Stress is part of our daily lives. Stress is a psychological state or process, by incidence of which a person is subject to events that are perceived as threatening to their physical and psychological well-being. Stress originates from interaction with the environment and occurs when there is a mismatch between the occasional pressures and the resources the person owns (Hossini, & Hossini, 2012). Evidence has shown that some degree of stress is necessary for success. However, high levels of stress follows several implications including physical and psychological diseases such as anxiety, depression, sleep disorders, restlessness, irritability, dementia, abnormal fatigue, reduced resistance and susceptibility to frequent infections, headache, reduced concentration, memory disorders, and decreased levels of problem-solving abilities (Craine et al., 2010).

One of the major stress-generating factors in everyone’s life is their occupation, where occupational stress has become a common and costly problem in occupational environments (Rhezaie et al., 2006). Occupational stress is the harmful physical and emotional responses of individuals occurring under conditions when the occupational requirements, abilities, and resources are incongruent with the needs of employees (Carlson et al., 2000). According to the control-demand model, occupational stress happens when the levels of psychological demands of a workplace are high, while the locus of control is limited in that job (Bourbonnais et al., 2001).

Occupational stress is one of the major problems of healthcare staff, substantially affecting the professional and personal performance of these individuals (Grzywacz et al., 2006). Occupational stress among healthcare staff is an important issue widely studied by researchers, considered as a physical and psychological threatening factor (Smith et al., 2005). In general, midwifery and nursing belong to high-stress jobs, where over 80% of the direct patients’ care is realized by these two major groups of health system. The results of various investigations have indicated that midwives suffer from high levels of occupational stress due to stress-generating factors in the delivery room including inappropriate physical environment, tolerance of pain and suffering, dealing with emergency cases of midwifery and the burden of responsibility for patients” health and alternating occupational shifts (Knezevic et al., 2011). In the study by Mohammadirizi et al., 58.7% of midwives suffered from severe occupational stress (Mohamadirizi et al., 2012). In the study by Anjazab and Farnia also more than 80% of midwives had experienced moderate to severe levels of occupational stress. Presence of occupational stress and, in turn, physical and psychological burnout results in leaving jobs, conflicts between the staff, health disorders, damage to occupational relations, reduced quality of cares, and occupational dissatisfaction (Enjezab & Farnia, 2002).

Although through elimination of stress is impossible, individuals can still learn its management. Relevant literature has regarded some interventions as effective to promote feelings of well-being together with adaptive mechanisms for reducing stress and risk of burnout (Alavi Arjmand et al., 2012). A review of the relevant literature has shown that training of skills of coping with stress as an enabler factor can be effective in mitigating the levels of stress and anxiety. In a review study on the impact of stress management, it was found that a combination of self-care programs including instruction of relaxation, social support, cognitive techniques, sports, and music is effective (Kravits et al., 2010). The study by Alavai Arjmand et al. (2012) demonstrated that stress management results in reduced level of occupational stress and job-life conflict. The important thing is sustainability of the developed changes. Through the cognitive behavioral variable, sustainable change would become possible (Alavi Arjmand et al., 2012). In the study by Hosseini et al., implementation of educational programs of stress management based on PROCEED model led to decreased levels of occupational stress in nurses in the intervention (Hosseini et al., 2011).

On the other hand, studies have shown that psychological-organizational damages as well as occupational pressures have a relationship with job satisfaction. Occupational pressures could have damaging effects on many of the variables related to performance due to irreversible damages to the mental health of individuals (Khayatan et al., 2013). Several studies including Weniggarden et al., Marzabadi and Tarkhorani have indicated that there is a significant negative relationship between occupational stress and satisfaction. Psychological burnout leads to job dissatisfaction, negative attitudes towards the self, the occupation, and life in general, and finally emergence of behaviors such as withdrawal from the job, absence, and quitting the job. In response to these pressures, the staff try to psychologically detach themselves from the job in a defensive manner in order to cope with the psychological pressures caused by the job. This leads them to become indifferent, suspicious, and rigid towards a job (Van Wijngaarden et al., 2004; Aliakbari Dehkordi et al., 2012; WHO, 1998).

Job satisfaction, which is known as a set of positive and negative attitudes of the self from their job, is influenced by many factors including salary and wages, relations, policies, procedures, occupational dimensions, jump discipline, and the personal characteristics of the staff (Rambur et al., 2003). Job satisfaction affects many positive organizational variables including increased productivity, the sympathy of staff towards the organization, their dedication and attachment to the work environment, increased work quantity and quality, proper relations, improved spirit, and interest towards the job (Hewitt et al., 2008). Job dissatisfaction in the hospital staff results in emotional detachment, indifference, and reduced quality of services offered to the patients. In contrast, their job satisfaction will be accompanied by improved care quality and increased productivity (Thyer, 2003).

Therefore, considering the occupational significance of midwives as one of the major components of healthcare system in order to offer services to two vulnerable strata of society, i.e. mothers and children, this study tries to investigate the effect of stress management on occupational stress and satisfaction of midwives employed in Obstetrics and Gynecology Hospital wards of Motahari Hospital in Jahrom, Iran.

## 2. Method

### 2.1 Identify Subsections

The present research was a Quasi-experimental pre- and post-clinical trial study; that was carried out after confirming by ethics committee of Jahrom University of medical science with number Jums.REC.1392.074.

Sample collection was conducted in the form of census including all of the Midwives in Obstetrics and Gynecology Hospital wards of Motahari Hospital in Jahrom, Iran.

### 2.2 Participant (Subject) Characteristics

The sample included 70 midwives who had worked at Obstetrics and Gynecology Hospital wards. Informed consent was obtained from the participants and they were assured that their information will remain confidential.

### 2.3 Sampling Procedures

The samples were divided into two 35-person groups of intervention and control. The intervention group participated in a two-day workshop of short-term training of stress management. The first session would involve introduction, familiarization, definition of a stress, causes, its symptoms and complications, familiarization with stress management, relaxation training, mental visualization, training of proper nutrition, and training of deep breathing. The second session would deal with anger management, determination, time management, recording daily events, discussion on saying “no”, delegation, conclusion, and questions and answers (Alavi Arjmand et al., 2012).

Next, two checklists of occupational stress and satisfaction would be completed right after the end of the workshop, second day, and one month after the workshop by the two intervention and control groups.

The instrument for data collection in this study included the checklist of demographic specifications -age, marital status-, occupational status -educational background, occupational background-, together with two questionnaires on occupational stress and occupational satisfaction. In order to measure occupational stress, the Toft-Anderson occupational stress questionnaire (1981) was used. This instrument contains 29 questions investigating the stress factors within 7 areas; pain and death of patients, six questions; conflict with physicians, 5 questions; lack of preparedness towards emotional needs of patients and their families, 2 questions; shortage of supportive resources, 3 questions; conflict with other colleagues and supervisors, 4 questions; workload, 5 questions; and lack of confidence in treatment, 4 questions. The responses are in the form of Likert scale beginning from never (0), sometimes (1), most of the time (2), and frequently (3) based on the perception of the respondent from the events of the work environment. According to the instruction guide of the instrument, acquiring a score between 0 and 28 signifies low stress, 29-57 shows moderate stress, and a score above 58 implies high levels of stress. In the study by Alavi Arjmand et al., the questionnaire’s validity was confirmed. Similarly, in the evaluation of reliability, the questionnaire was also verified through calculation of a Cronbach alpha coefficient of 0.78 (Alavi Arjmand et al., 2012). In a similar vein, in the study by Payami et al, the questionnaire enjoyed a reliability of 0.85 (Mehrabi et al., 2010).

In order to investigate occupational satisfaction, JDI checklist of Stephen Robbins (1994) was utilized. This questionnaire contains 39 items of 5-option Likert scale type in which the satisfaction of employees are examined within five dimensions of satisfaction from the job (10 questions), satisfaction from the supervisor (8 questions), satisfaction from colleagues (8 questions), promotion (4 questions), and payment (5 questions). A total score of 0-64 indicated low satisfaction, 65-129 showed moderate satisfaction, and 130-195 revealed high satisfaction. The questionnaire’s reliability was investigated by Ranjbar (2005) who obtained a Cronbach alpha of 0.73. Similarly, Bambaei Ro (2006) obtained a reliability of 0.72 using the replication method. In order to determine the internal reliability, Cronbach alpha method was reused culminating in a value of 0.83 (Moghimi, 1998).

The participants in this study were ensured that the collected information would remain confidential. In order to analyze the information, SPSS 16 together with descriptive statistics tests (frequency, percentile, mean, and standard deviation), sample t-test, and Spearman correlation coefficient were used.

## 3. Results

A total number of 70 midwives participated in this study. The total mean of samples’ age was 31.72±7.38 years of age. T-test indicated that there is no significant difference between the age average of the control group (27.74±5.28) and that of the intervention (29.75±7.07) (p=0.086). The overall average of the midwives’ background was 7.07±6.72 years. There was a significant difference between the average work experience of the control group (6.20±2.99) and that of the intervention (10.94±7.19), according to independent sample t-test (p=0.000). From among the individuals, 25 (35.2%) were single and 45 (63.4%) were married. In terms of the type of job, 17 (23.9%) were contractual, 16 (22.5%) were plan-based, and 37 (52.1%) were employed as covenant and official employees. The following tables indicate a comparison of the mean score of occupational satisfaction and occupational stress at three times of before, right after, and one month after the intervention.

**Table 1:** comparison of the mean score of occupational satisfaction in the two groups of intervention and control: before, right after, and one month after the intervention

Groups	Mean (SD) (Before)	Mean (SD) (After)	Mean (SD) (1 month after)
**Control**	113.03(24.97)	111.03(24.97)	102.46(32.17)
**Intervention**	123.94(32.17)	126.03(26.26)	111.83(32.46)
**Sum**	118.49(29.11)	119.53(26.26)	107.14(35.02)
**F**	0.086		

**p- value**	NS		

The findings of the above table indicated that although in the intervention group, the mean score of satisfaction has increased right after the stress management workshop, based on repeat measurement tests, no significant difference was observed between the mean score of satisfaction before, after, and one month after the intervention across the two groups. Furthermore, based on the independent sample t-test, no significant difference was seen between the score of job satisfaction across the two groups before the intervention (p=0.271).

**Table 2:** Comparison of the mean score of occupational stress in the two groups of intervention and control: before, right after, and one month after intervention

Groups	Mean (SD) (Before)	Mean (SD) (After)	Mean (SD) (1 month after)
**Control**	29.97(9.13)	29.97(9.13)	29.04(8.6)
**Intervention**	38.37(11.96)	31.60(11.93)	37.14(9.9)
**Sum**	30.78(10.58)	32.94(12.35)	31.07(11.30)
**F**	11.645		
**p- value**	0.001		

The findings of the above table reveal that based on repeated measurement test, there is a significant difference between the mean scores of occupational stress between, right after, and one month after holding the workshop across the two groups of intervention and control. Based on independent sample t-test, there was no significant difference between the before intervention and one month after intervention (p=0.083) in terms of occupational stress. However, right after the intervention, a significant difference was observed between the mean scores of stress across the two groups (p=0.004).

Based on Pearson correlation coefficient, there was a significant relationship between occupational stress and occupational satisfaction of all of the three stages in this study (p=0.000).

## 4. Discussion

The findings of this study revealed that holding the stress management workshop resulted in mitigation of occupational stress right after the workshop. This, however, had no significant effect on job satisfaction by midwives. Considering the effect of stress management program on reduction of stress, the findings of this study are congruent with them (Rezaei et al., 2011; Hamid et al., 2014; Eli et al., 2014; Kim et al., 2014). In the study by Khadivzadeh et al (2014), training of stress management resulted in diminished psychological and occupational problems of the staff (Khadivi et al., 2014). In the study by Zamani et al. training of stress management resulted in diminished levels of anxiety and depression in the patients suffering from multiple sclerosis (Zamani et al., 2014). Stress management aims at helping individuals through four stages to re-create their cognitions: 1. Awareness 2. Re-evaluation of the situation 3. Acceptance and substitution and 4. Evaluation of the new mindset (Kraimer, 2014). Stress management intervention targets the thoughts of an individual as a cognitive process. The basis of this theory is that alteration of behaviors and emotions is determined by the thoughts about the happened events. Individuals are often worried and distressed by their perception of events rather than the events themselves. The self-management skills and psychological preparedness in the form of stress management held the individuals manage the stress in the workplace in an effective way and thus have a better productivity in their jobs (Seibert et al., 2013). Occupational stress results in gradual development of mental beer now and lowered energy levels in the individual (Espeland, 2006). Stress management skills bring about continuation of daily activities, previously stopped on the influence of the stressor through establishing an emotional balance, helping the individual to feel mentally and psychologically empowered (Kang, 2013).

In this study, right after the workshop, the levels of occupational stress reduced. However, after one month, it returned to its initial state. In the programs and the strategies of stress management, a stress operating in a causal relationship, if its cause or causes are not eliminated, the degree of effect of stress control programs would also be temporary (Price, 2011). In a study on 147 midwives, the findings indicated that numerous social-demographic factors including work background, workplace, the extent of passion towards the occupation, and the educational background level of individuals have permanent effects on the amount of occupational stress (Alparslan, & Doganer, 2009). Sustainability of the developed changes is of paramount importance in planning for stress management. Through behavioral and cognitive variables, sustainable change is possible (Alavi Arjmand et al., 2012), since the multidimensional nature of stress requires all-round preparedness in the workplace to manage stress (Bianchi, & Milkie, 2010). In the study by Hirokawa et al., a three-hour stress management program including training of relaxation and determination in hospital staff resulted in temporary mitigation of occupational stress. Sustainability of the state has proven to be contingent upon continuation of stress management program and application of other methods in addition to removing or declining stressor factors (Hirokawa et al., 2012). In another research, stress management training led to decreased levels of occupational stress in nurses (Moeini et al., 2011). Midwives, based on their occupational nature have to deal with high levels of stress in their work environment. Coping with these conditions necessitates application of all individual and organizational capacities. Use of temporary strategies would not have a sustainable effect in this area (Mollart et al., 2013).

In this research, stress management program did not have a significant effect on job satisfaction by midwives. In the study by Skinner et al. on 538 midwives, the findings indicated that the levels of job satisfaction were influenced by factors related to the workplace, spiritual-social factors and the stress present in the workplace (Skinner et al., 2007).

The findings of this research indicated that there is a significant relationship between occupational stress and satisfaction, congruent with the results of earlier researches (Bazkum et al., 2013; Drury et al., 2014; An et al., 2013).

Due to their stressful occupational nature, midwives should develop their adaptation and interpersonal relationship scales when coping with stressful work conditions.

Use of questionnaires was one of the limitations of this research. The limitations related to this instrument should be, therefore, taken into consideration. For example, despite the emphases and required explanations by the executor, it might be possible that some individuals refrain from presenting real responses and might have given superficial and careless answers.

It is suggested that psychologists and psychiatrists help the staff in coping with occupational stress by developing educational methods, courses, as well as different and periodic workshops considering the proof of effectiveness of stress management training and stressful work conditions and midwifery wards.

since the costs related to occupational stress can be highly significant, and as several research has shown that emergence of occupational stress and dissatisfaction in stressful environments has a direct relationship with damages, absence, reduced productivity, increased errors, and emergence of abnormal behaviors by the staff, it seems that closer attention to this issue by planners, officials, and managers would result in reduced costs as well as direct and indirect implications and thereby promoted health of the society.
